# Time course of electrocortical food-cue responses during cognitive regulation of craving

**DOI:** 10.3389/fpsyg.2013.00669

**Published:** 2013-09-30

**Authors:** Adrian Meule, Andrea Kübler, Jens Blechert

**Affiliations:** ^1^Department of Psychology I, Institute of Psychology, University of WürzburgWürzburg, Germany; ^2^Division of Clinical Psychology, Psychotherapy and Health Psychology, University of SalzburgSalzburg, Austria

**Keywords:** food craving, food-cues, calorie content, eating, EEG, event-related potentials, LPP, slow wave

## Abstract

In our current obesogenic environment, exposure to visual food-cues can easily lead to craving and overeating because short-term, pleasurable effects of food intake dominate over the anticipated long-term adverse effects such as weight gain and associated health problems. Here we contrasted these two conditions during food-cue presentation while acquiring event-related potentials (ERPs) and subjective craving ratings. Female participants (*n* = 25) were presented with either high-calorie (HC) or low-calorie (LC) food images under instructions to imagine either immediate (NOW) or long-term effects (LATER) of consumption. On subjective ratings for HC foods, the LATER perspective reduced cravings as compared to the NOW perspective. For LC foods, by contrast, craving increased under the LATER perspective. Early ERPs (occipital N1, 150–200 ms) were sensitive to food type but not to perspective. Late ERPs (late positive potential, LPP, 350–550 ms) were larger in the HC-LATER condition than in all other conditions, possibly indicating that a cognitive focus on negative long-term consequences induced negative arousal. This enhancement for HC-LATER attenuated to the level of the LC conditions during the later slow wave (550–3000 ms), but amplitude in the HC-NOW condition was larger than in all other conditions, possibly due to a delayed appetitive response. Across all conditions, LPP amplitudes were positively correlated with self-reported emotional eating. In sum, results reveal that regulation effects are secondary to an early attentional analysis of food type and dynamically evolve over time. Adopting a long-term perspective on eating might promote a healthier food choice across a range of food types.

## Introduction

High-calorie (HC) foods and food-cues are ubiquitous in western or westernized societies. Those stimuli exert a strong influence on eating behavior, e.g., initiate eating or lead to an increased food intake in an automatic and implicit fashion (Cohen and Farley, 2008; Cohen and Babey, 2012). Thus, a constant self-monitoring and -regulation of eating behavior is necessary to avoid indulging in eating palatable, high caloric foods (Lowe, 2003). Food- and food-cue exposure trigger so-called cephalic phase responses that prepare the organism for the consumption of food and are associated with an increase in craving for those foods (Nederkoorn et al., 2000; Legenbauer et al., 2004; Rodríguez et al., 2005). Likewise, neuroimaging studies have shown that presentation of visual food-cues markedly activate the human brain, particularly subcortical areas associated with reward and incentive salience (Wang et al., 2004; Kenny, 2011; Carnell et al., 2012; García-García et al., 2013). Accumulating evidence suggests that those food-cue induced subcortical activations can be downregulated by the use of cognitive strategies, probably through increased inhibitory signals from prefrontal cortices (Wang et al., 2009; Kober et al., 2010b; Hollmann et al., 2012; Scharmüller et al., 2012; Siep et al., 2012; Yokum and Stice, 2013).

Yet, little is known about the time course of neural activity supporting such modulations. It would be useful to know, however, whether successful regulation extends to earlier, more implicit evaluative processes or is limited to later processing stages. Event-related potentials (ERPs) afford the temporal resolution to distinguish multiple early, mid-latency, and late components related to appetitive processing. In affective picture viewing, early (e.g., N100, 100–200 ms) and mid-latency ERPs [e.g., early posterior negativity (EPN), 200–300 ms] reflect physical stimulus factors but may also index early selective attention (Olofsson et al., 2008). In the food context, Toepel et al. (2009) showed that pictorial low-calorie (LC) food-cues elicited a larger relative negativity as compared to HC food-cues after ~150–200 ms at occipital electrodes. Thus, this study complements imaging studies showing that HC and LC food-cues are differently processed in the brain (Killgore et al., 2003; Siep et al., 2009; Frank et al., 2010) and that this discrimination occurs automatically and rapidly. Also mid-latency ERPs such as the EPN are of interest in this context as it has been shown that the EPN is sensitive to food deprivation (Stockburger et al., 2008) and eating disorder status (Blechert et al., 2011).

Long latency ERPs (>300 ms) index maintained attention, memory storage or meaning evaluation (Schupp et al., 2006; Hajcak et al., 2010) and are subject to cognitive (top–down) modulations (Olofsson et al., 2008). A positive, centro-parietal ERP component beginning at ~300 ms after stimulus onset is known as the P300 or Late Positive Potential (LPP). The LPP has been found to be enhanced in response to highly arousing stimuli, e.g., positive and negative pictures (Olofsson et al., 2008; Hajcak et al., 2010). Thus, it has been proposed that the LPP indicates *motivated attention* toward stimuli that are evolutionary relevant as they automatically attract attention and appear to be dependent on motivational factors such as approach or avoidance tendencies (cf. Littel et al., 2012). The LPP is also increased in response to substance-related compared to neutral cues in substance users (Littel et al., 2012).

Likewise, the LPP seems to reflect the motivational value of food stimuli and is modulated by food deprivation and individual differences in eating behavior. Nijs et al. (2008) found that food pictures elicited an enlarged LPP as compared to pictures of neutral objects. Moreover, increased LPP amplitude was found in response to food pictures when participants were hungry as compared to when they were satiated (Stockburger et al., 2009b; Nijs et al., 2010). With regard to individual differences, elevated LPP amplitude in response to food pictures was found in external eaters (Nijs et al., 2009), women with binge eating disorder (Svaldi et al., 2010), and emotional eaters (Blechert et al., in revision). However, no differences in food-related LPP amplitude could be observed between normal-weight vs. obese participants (Nijs et al., 2008) and high chocolate cravers vs. low chocolate cravers (Asmaro et al., 2012). In another study, the LPP in response to food pictures did not differ from neutral pictures, but was attenuated in restrained eaters when foods were available for direct consumption (Blechert et al., 2010). To summarize, most studies found that the LPP is enlarged in response to food pictures as compared to neutral pictures, particularly when participants were hungry. Some studies also point out that an enhanced food-related LPP is associated with habitual overeating and related measures, but results are not conclusive yet.

Whereas the LPP appears to be transient, a later slow wave is typically enhanced for several seconds after presentation of motivationally relevant stimuli. It has been argued that the LPP and slow wave are functionally similar and, thus, the slow wave may reflect additional attentive processing or a continuation of attentive processing of motivationally relevant stimuli (Littel et al., 2012). Both the LPP and the slow wave are subject to cognitive modulation. Several affective picture viewing studies demonstrated reductions in amplitudes during cognitive emotion regulation strategies such as distraction or reappraisal (cf. Hajcak et al., 2010). Moreover, time course of LPP/slow wave modulations to negative images depended on the specific emotion regulation strategy used: distraction led to an earlier attenuation of the LPP than reappraisal, possibly due to the more effortful processing in the latter (Thiruchselvam et al., 2011).

However, down-regulation of arousing material does not uniformly reduce LPP amplitudes. Other studies found the LPP to be enlarged during instructions to decrease emotions as compared to passively viewing emotional pictures (Langeslag and Van Strien, 2010; Baur et al., submitted). A similar pattern was found by Littel and Franken (2011), who investigated craving regulation strategies in smokers while watching smoking and neutral pictures. Passively viewing smoking pictures elicited larger LPP amplitudes as compared to watching neutral pictures. However, unexpectedly, reappraisal strategies did not attenuate the LPP in response to smoking pictures. Only when distinguishing different cognitive strategies they found that distraction strategies (thinking about something different) reduced the LPP after ~1 s. This suggests that modulation of later LPP stages may depend on the specific type of reappraisal that is applied.

In the current study, we investigated if cognitive strategies for modulating food craving would alter ERPs in response to pictorial food-cues. Specifically, we adapted the paradigm used in the study by Kober et al. (2010a,b) in which participants should either focus on the long-term consequences or the immediate consequences of eating HC foods. We expected that focusing on the long-term effects would *decrease* food craving for those foods as compared to focusing on the immediate effects (Kober et al., 2010a,b). As a control condition, we presented pictures of LC food items with the very same instructions. Here, we expected that thinking about the long-term effects (e.g., health benefits) would *increase* craving for those foods as compared to focusing on immediate consumption.

With regard to ERP analyses, we aimed at replicating general, i.e., perspective independent, differences in ERP amplitudes between HC and LC food pictures. Specifically, we tested if there would be an elevated negativity in response to LC foods as compared to HC foods in an early time window (150–200 ms) at occipital sites as reported by Toepel et al. (2009). Our predictions for effects of perspective (long-term vs. short-term) focused on HC images for which we expected reduced craving and LPP amplitudes under a long-term perspective based on findings from emotion regulation research (e.g., Hajcak et al., 2010; Thiruchselvam et al., 2011). Perspective might also modulate ERP responses to LC images but predictions were less clear here. In accordance with prior studies on emotion and craving regulation (Littel and Franken, 2011; Thiruchselvam et al., 2011), we divided the LPP into an earlier (350–550 ms) and later component (550–3000 ms, slow wave) as some effects of reappraisal on LPP/slow wave amplitudes may only be observed in later stages of food-cue processing. Finally, we explored if trait measures of eating behavior (food cravings, restrained eating, external eating, emotional eating, and eating disorder symptomatology) and state food cravings would be correlated with ERP amplitudes.

## Materials and methods

### Participants

Twenty-six female Psychology students of the University of Salzburg, Austria, participated in exchange for course credit or €10. Exclusion criteria were presence of cardiovascular or neurological diseases, diabetes, regular use of medication other than contraceptives, age <18 or >30 years, and underweight (BMI < 17.50 kg/m^2^) or obesity (BMI ≥ 30.00 kg/m^2^). Vegetarians were also excluded because of altered attentional processing (as indicated by the LPP) of meat dishes (Stockburger et al., 2009a). Descriptive statistics of participant characteristics are depicted in Table 1. Mean scores on the eating behavior measures were comparable to scores in other non-clinical samples (e.g., Hilbert et al., 2007). Importantly, none of our participants scored above the cutoff for clinical eating disorders (i.e., a total score of at least 4 on the *Eating Disorder Examination—Questionnaire*; Carter et al., 2001; Mond et al., 2006). Half of the sample (*n* = 13) reported to be currently dieting (see section Dieting Status). Dieters did not differ from non-dieters in craving ratings [all *t*s_(24)_ < 0.98, *ns*] and ERP amplitudes [all *t*s_(23)_ < 0.82, *ns*].

**Table 1:** **Descriptive statistics of participant characteristics**.

***N* = 26**	***M***	***SD***	**Range**
Age (years)	23.00	2.23	18.00–27.00
Body-mass-index (kg/m^2^)	23.12	2.80	17.60–27.80
Food deprivation (h)	3.49	0.60	2.30–4.30
Last meal (kcal)	487.54	245.59	101.52–990.80
Food Cravings Questionnaire—Trait	108.15	21.08	74.00–147.00
Food Cravings Questionnaire—State			
Before task	27.62	8.86	15.00–51.00
After task	43.15	9.04	30.00–65.00
Eating Disorder Examination—Questionnaire	1.23	0.97	0.19–3.88
Dutch Eating Behavior Questionnaire		
Restrained eating	2.58	0.73	1.10–4.60
Emotional eating	2.28	0.57	1.50–3.70
External eating	3.42	0.68	2.00–4.80
Restraint Scale	13.77	5.93	5.00–26.00

### Questionnaires

#### Food cravings questionnaires (FCQ)

The trait version of the FCQ (FCQ-T; Cepeda-Benito et al., 2000) consists of 39 items and assesses the frequency of food craving experiences on nine subscales (intentions and plans to consume food, anticipation of positive reinforcement, anticipation of relief from negative states, lack of control over eating, thoughts, or preoccupation with food, hunger, emotions before or during food cravings, cue-dependent food cravings, and guilt). Only the total score was used in the current study. Internal consistency of the German version is α = 0.96 (Meule et al., 2012a) and was α = 0.91 in the current study.

The state version of the FCQ (FCQ-S; Cepeda-Benito et al., 2000) consists of 15 items and measures current food craving on five subscales (intense desire to eat, anticipation of positive reinforcement, anticipation of relief from negative states, lack of control over eating, and hunger). Only the total score was used in the current study. Internal consistency of the German version is α = 0.92 (Meule et al., 2012a). Participants completed the FCQ-S at the beginning and at the end of the testing session and internal consistency was α = 0.90 (before) and α = 0.83 (after) in the current study.

#### Eating disorder examination – questionnaire (EDE-Q)

The EDE-Q (Fairburn and Beglin, 1994) consists of 22 items and assesses eating disorder psychopathology on four subscales (restraint, eating concern, weight concern, and shape concern). Only the total score was used in the current study. Internal consistency of the German version is α = 0.97 (Hilbert et al., 2007) and was α = 0.94 in the current study.

#### Dutch eating behavior questionnaire (DEBQ)

The DEBQ (van Strien et al., 1986) consists of 30 items and measures three aspects of eating behavior (restrained eating, external eating, and emotional eating). Internal consistencies of the German version are α > 0.80 for all three subscales (Grunert, 1989) and ranged between α = 0.83–0.88 in the current study.

#### Restraint scale (RS)

The RS (Herman and Polivy, 1980) consists of 10 items and assesses restrained eating behavior on two subscales (concern for dieting and weight fluctuations). Only the total score was used in the current study. Internal consistency of the German version is α = 0.83 (Dinkel et al., 2005) and was α = 0.82 in the current study.

#### Dieting status

Current dieting status (yes/no) was assessed with a single question [“Are you currently restricting your food intake to control your weight (e.g., by eating less or avoiding certain foods)?”; cf. (Meule et al., 2012b)].

### Stimuli

**Figure 1:**
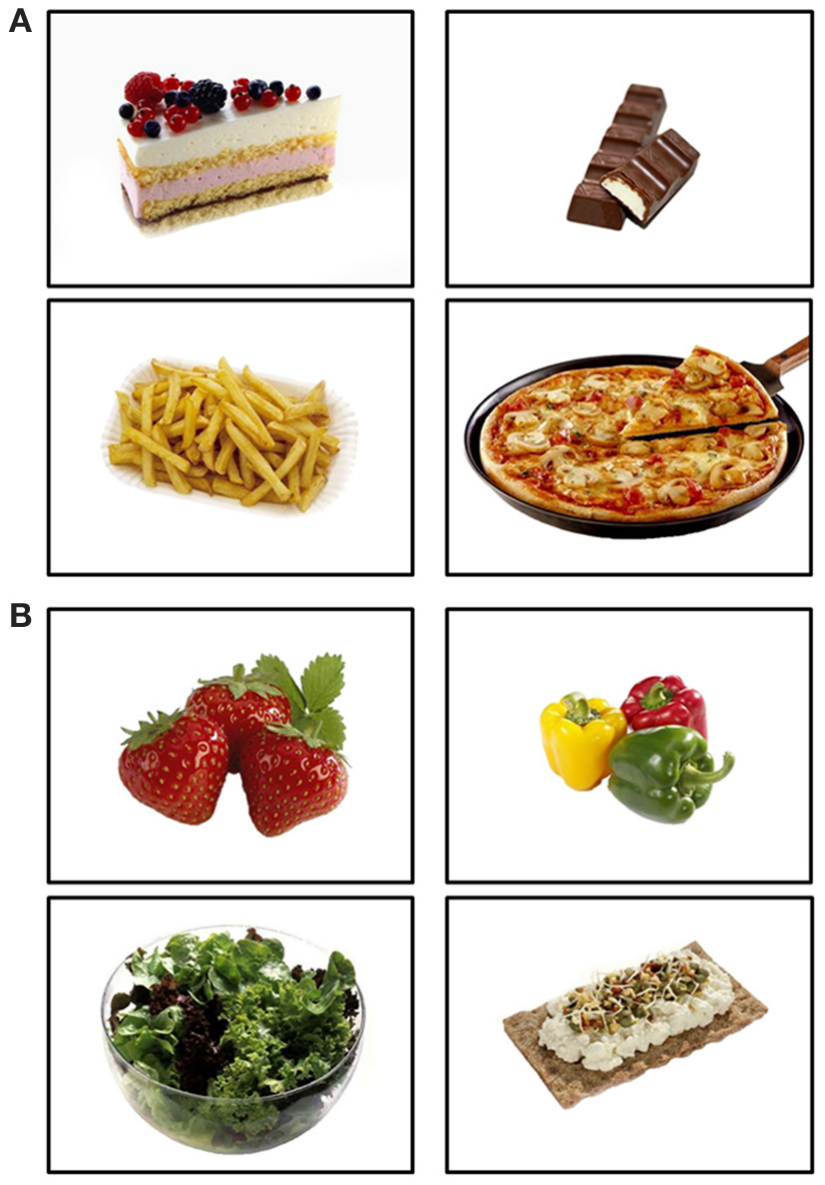
**Examples of high-calorie (A) and low-calorie (B) food pictures**.

Stimuli were selected from a food picture database featuring food images with simple figure ground compositions for experimental research (Meule and Blechert, 2012; also see www.food-pics.sbg.ac.at) and comprised pictures of 34 HC and 34 LC foods. HC food pictures included both sweet and savory foods (Figure 1A, Table 2). LC food pictures included vegetables, fruits, salad, and crisp bread (Figure 1B, Table 2). All pictures had the same resolution and color depth (600 × 450 pixels, 96 dpi, 24 bpp) and were homogenous with regard to background color and camera distance. HC and LC food pictures did not differ in RGB brightness and contrast [all *t*s_(66)_ < 0.78, *ns*], visual complexity [jpg compression: *t*_(66)_ = −0.48, *ns*; edge detection: *t*_(66)_ = −0.95, *ns*; subjective complexity ratings: *t*_(66)_ = −1.41, *ns*]. The food picture database also includes subjective palatability ratings from a sample of young, female students (Meule and Blechert, 2012). Analyses of those ratings showed that palatability did not differ between HC and LC food pictures [*t*_(66)_ = 1.36, *ns*]. HC food pictures displayed foods with a higher calorie density (*M* = 360.98 kcal/100g, *SD* = 140.87) as compared with LC food pictures [*M* = 35.44 kcal/100g, *SD* = 26.66; *t*_(66)_ = 13.24, *p* < 0.001]. Similarly, the total amount of calories displayed in HC food pictures (*M* = 625.23 kcal/image, *SD* = 680.92) was higher than that of LC food pictures [*M* = 97.95 kcal/image, *SD* = 109.81, *t*_(66)_ = 4.56, *p* < 0.001].

**Table 2:** **List of foods pictures of which were used in the current study**.

	**Low-calorie foods(#)**	**High-calorie foods(#)**
Practice block	Blueberries (248)	Chips (043)
	Grapes (281)	Chocolate chips (174)
	Paprika peppers (239)	Cream cake (028)
	Pears (241)	Pizza with mushrooms (032)
	Strawberries (234)	Popsicle with chocolate and nuts (116)
Main task	Apples (238)	Bavarian doughnut (178)
	Blueberries (202)	Bundt cake (096)
	Carrots (208)	Butter (064)
	Cauliflower (249)	Candies (102)
	Celery (262)	Cashew nuts (110)
	Cherries (280)	Cheeseburger with French fries (003)
	Crisp bread with cottage cheese (205)	Cheesecake with cherries (001)
	Cucumber (267)	Cheesecake with cherries (136)
	Cucumber and carrots (215)	Cheesecake with strawberries (006)
	Fennel (277)	Chocolate bar (173)
	Figs (254)	Chocolate cookie (004)
	Grapefruit (256)	Chocolate covered nuts (160)
	Grapes (284)	Chocolate crisps (165)
	Kiwi (194)	Chocolate croissant (184)
	Lettuce (232)	Chocolate marshmallows (166)
	Limes (269)	Chocolate marshmallows (161)
	Mixed berries (203)	Chocolate muffins (048)
	Mixed berries (209)	Cream cake with raspberries (055)
	Mushrooms (263)	French fries (046)
	Nectarines (216)	Fruit gum (153)
	Oranges (200)	Ham and cheese sandwich with chips (057)
	Paprika peppers (198)	Lollipops (123)
	Pineapple (285)	Lollipops (124)
	Pomegranate (255)	Meatballs (190)
	Radishes (258)	Pancakes with syrup (016)
	Raspberries (206)	Pasta bake (143)
	Red cabbage (259)	Pizza with vegetables (131)
	Salad onions (266)	Salami (176)
	Strawberries (243)	Scoops of ice cream (038)
	Tomatoes (275)	Slice of bread with chocolate hazelnut spread (189)
	Turnip cabbage (268)	Spaghetti with tomato sauce (010)
	Vegetables with dip (212)	Spritz cookies (148)
	Water melon (199)	Strawberry cake (089)
	Zucchini (265)	Sugar-glazed doughnut (041)

^#^Refers to the picture number in the food-pics database, see www.food-pics.sbg.ac.at

### Regulation of craving (ROC) task

**Figure 2:**
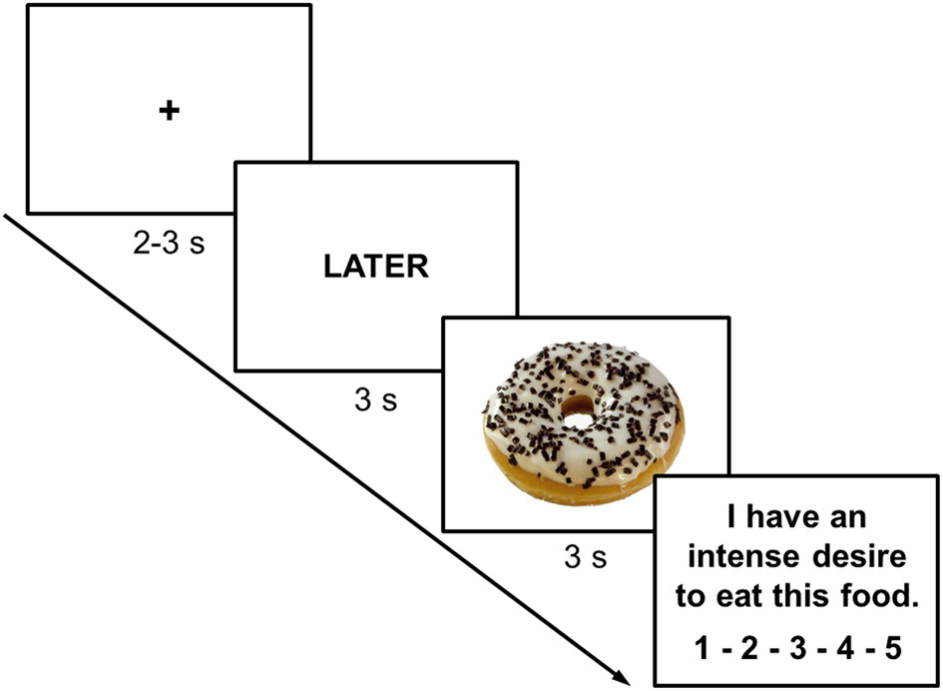
**Representative screen displays of a trial in which participants were instructed to think of the long-term consequences of eating a high-calorie food**. Inter-trial interval (fixation cross) was jittered between 2000 and 3000 ms while instruction cues and food-cues were presented for 3000 ms each. At the end of each trial participants rated their current craving for the presented food on a 5-point scale from *not at all to very strong*.

The ROC-task was adapted from the task by Kober and colleagues which involved smoking-related cues (Kober et al., 2010a,b). The task was programmed with E-prime 2.0 (Psychology Software Tools Inc., Pittsburgh, PA) and displayed on a 23″ LCD-monitor with a resolution of 1920 × 1080 pixels at 120 Hz. One experimental trial started with a fixation cross (duration varying randomly between 2000 and 3000 ms). Then, a cue was presented for 3000 ms instructing participants to either focus on the immediate (NOW) or long-term (LATER) consequences of eating the food item presented on the following slide. Either a high-caloric (HC) or a low-caloric (LC) food item was then presented for 3000 ms (Figure 2). Finally, participants indicated their current craving (“I have an intense desire to eat this food.”) on a 5-point scale from *not at all* to *very strong* (Figure 2). Participants first performed a practice block including 8 trials (2 LC-NOW, 2 HC-NOW, 2 LC-LATER, 2 HC-LATER) with 5 HC food pictures and 5 LC food pictures that were not used in the experimental task. The experimental task consisted of 136 trials with a short break after half of the trials. Each participant viewed 34 HC and 34 LC food pictures preceded by either the NOW or the LATER instruction (17 LC-NOW, 17 HC-NOW, 17 LC-LATER, 17 HC-LATER) in pseudorandomized order. The very same set was repeated after the break. Across participants, counterbalancing ensured that each picture was shown in the NOW vs. LATER conditions with equal probabilities.

### Apparatus

EEG was recorded with an actively shielded 65-channel electrode cap (sintered Ag/AgCl electrodes, manufactured for TMS International, Oldenzaal, the Netherlands). All scalp positions of the International 10–20 System were used, with additional sites 10% inferior to the standard electrodes (PO9, TP9, FT9, PO10, TP10, FT10). Data were recorded with a REFA 8–72 digital amplifier system (TMSi, Oldenzaal, the Netherlands) at a sample rate of 512 Hz and 24 bit/channel and were filtered online at 0.05–100.00 Hz. The unipolar inputs were configured as a reference amplifier: All channels were amplified against the average of all connected inputs. A wet band on the left wrist was used as patient ground. Vertical EOG was recorded with bipolar electrodes above and below the right eye. Electrode impedances were kept below 50 kOhms for all the electrodes which is appropriate for this type of high-input impedance amplifier (Ferree et al., 2001). Data acquisition was controlled through TMSi Polybench (TMSi, Oldenzaal, Netherlands).

Data were analyzed offline with Brain Vision Analyzer 2.0 (Brain Products GmbH, Gilching, Germany) and comprised the following steps: Low pass filtering at 20 Hz, semi-automatic eye-blink correction using independent component analysis, manual screening for remaining artifacts or bad channels, segmentation (200 ms baseline, 3000 ms picture), artifact correction (Exclusion of Epochs exceeding >150 μV amplitude change or low activity), baseline-subtraction (200 ms) und averaging of segments for each experimental condition (LC-NOW, HC-NOW, LC-LATER, HC-LATER). EEG data of one participant were excluded due to technical problems. Overall number of valid segments was high (95.91%) and did not differ by condition [*F*_(3, 72)_ = 1.30, *ns*].

### Procedure

Participants completed the trait-related questionnaires online, a few days prior to the experiment in order to avoid a possible influence of performing the ROC task on questionnaire scores. On the day of the testing session, all participants ate lunch between 12 and 13 p.m. and were asked to refrain from eating until the experiment to obtain roughly comparable levels of satiety at the time of testing. All participants were tested between 3 and 4:30 p.m. On arrival at the laboratory, participants provided written informed consent and weight and height was measured. After set-up of the psychophysiological equipment, participants completed the FCQ-S. All participants were fully aware of the fact that the foods presented it the current study would not be available to eat in the laboratory during or after the experiment.

Prior to the ROC task, participants underwent a structured training session which was adapted from the instructions by Kober et al. (2010a,b). During this session, participants were trained to focus on the odor, taste, and consistency of the presented food during eating after the NOW-instruction. After the LATER-instruction, participants were instructed to think of, e.g., change in body weight that would be associated with frequent consumption of the presented food item and of other health consequences. Participants then performed a self-paced practice block under the experimenter's supervision before the main task commenced.

After the ROC-task, participants completed the FCQ-S again and were asked to rate their overall success in following instructions in percent (i.e., 100% represent a subjectively perceived success in regulating craving).

### Data analysis

Craving ratings were averaged across the 34 trials per condition. Craving ratings and ERP voltage measures were submitted to 2 (perspective) × 2 (picture type) analyses of variance (ANOVAs) for repeated measures. *Post-hoc t*-tests were calculated to follow up on interaction effects. Changes in state cravings during the task were captured in a difference score between FCQ-S before vs. after the ROC-task and submitted to correlational analyses (see below). Scores on the FCQ-S before and after the task were also compared using paired *t*-test.

Based on the findings by Toepel et al. (2009), we investigated if HC and LC food pictures elicited differential ERP amplitude at posterior sites in an early time window independent of perspective. For this purpose, we visually explored difference waveforms for HC minus LC food pictures. This difference was maximal during a relative negativity at occipital sites (PO9, PO7, PO3, POz, PO4, PO8, PO10, O1, Oz, O2) between 150 and 200 ms (N1, see below).

**Figure 3:**
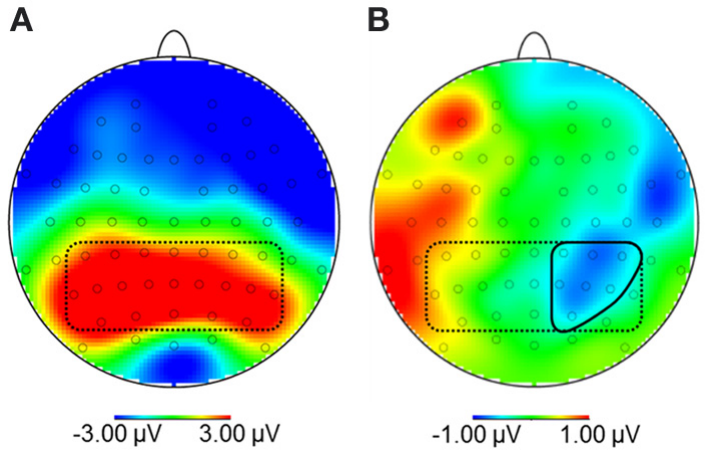
**(A)** Topography of maximal amplitudes of the Late Positive Potential between 350 and 550 ms (encircled by dotted line). **(B)** Within this region, sensors where the difference for high-calorie food pictures after the NOW vs. LATER perspective was maximal were collapsed (CP2, CP4, CP6, P2, P4, P6, PO4; encircled by solid line).

Due to the broad distribution and variation in amplitude maximum of the LPP in food image and affective picture viewing paradigms (e.g., Littel et al., 2012), we adopted a two-step localization approach. First, visual inspection of grand averages was used to determine the timing and location of the LPP maximum. Second, we calculated a difference waveform HC-NOW minus HC-LATER which we evaluated within this region, to determine those electrodes sites where regulation affected the LPP. Step one revealed that the LPP was maximal on bilateral centro-parieto-occipital electrodes between 350 and 550 ms (see Figure 3A). Time and region is thus consistent with several other reports on food image processing (Blechert et al., 2010; Nijs et al., 2010; Svaldi et al., 2010; Asmaro et al., 2012). Step two revealed that within this broad region, perspective effects were lateralized predominantly to the right hemisphere and, thus, sensors CP2, CP4, CP6, P2, P4, P6, PO4 were collapsed (Figure 3B) for statistical analysis. Based on the finding that differences between conditions shift over the time course in later stages of the LPP/slow wave when cognitive craving regulation strategies are used (Littel and Franken, 2011), we followed up the progression of the LPP in the very same cluster between 550 and 3000 ms (slow wave).

Differences in ERP amplitudes (μV) between conditions were tested with 2 (perspective) × 2 (picture type) ANOVAs for repeated measures. Finally, we calculated correlations between ERP amplitudes for each condition and subjective craving ratings as well as all eating behavior questionnaires. Results were considered as significant at an α level of *p* = 0.05. Results marked as *ns* refer to *p*-values > 0.05.

## Results

### Craving and performance ratings

**Figure 4:**
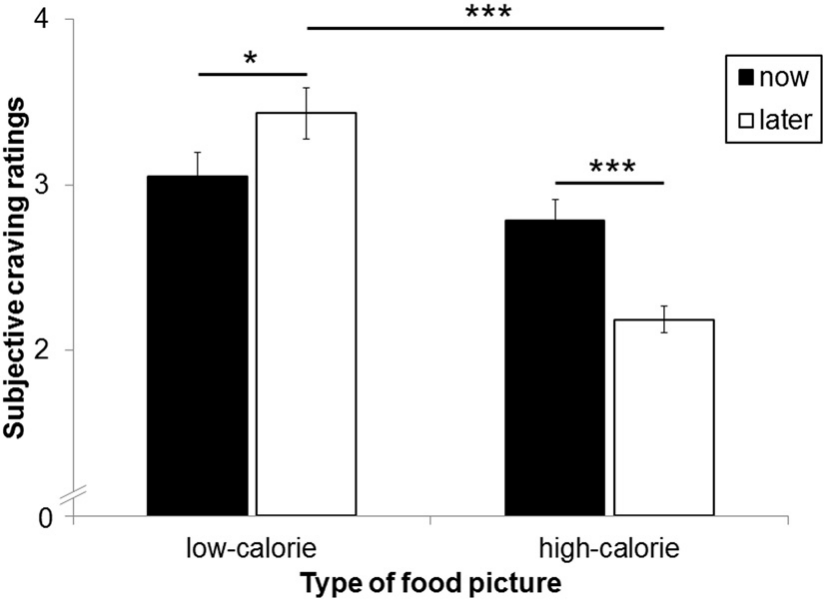
**Mean craving ratings after each food picture as a function of calorie content and perspective type**. Error bars represent standard errors. Asterisks indicate ^*^p < 0.05 and ^***^p < 0.001.

The main effect for perspective on craving ratings was not significant [*F*_(1, 25)_ = 1.74, *ns*]. A significant main effect for picture type [*F*_(1, 25)_ = 31.11, *p* < 0.001, η^2^_*p*_ = 0.55] was modulated by perspective [interaction perspective × picture type: *F*_(1,25)_ = 13.85, *p* < 0.01, η^2^_*p*_ = 0.36; Figure 4]. As expected, craving for HC food pictures was rated higher after the NOW (*M* = 2.78, *SD* = 0.67) as compared to the LATER perspective [*M* = 2.19, *SD* = 0.42, *t*_(25)_ = 4.35, *p* < 0.001]. For LC food pictures, by contrast, craving ratings were higher after the LATER (*M* = 3.43, *SD* = 0.79) compared to the NOW perspective [*M* = 3.05, *SD* = 0.76, *t*_(25)_ = 2.26, *p* < 0.05]. Craving for HC and LC foods did not differ after the NOW perspective [*t*_(25)_ = 1.33, *ns*], but were higher for LC foods compared to HC foods after the LATER perspective [*t*_(25)_ = 7.03, *p* < 0.001].

Overall craving ratings (i.e., FCQ-S scores) were higher after the ROC-task (*M* = 43.15, *SD* = 9.04) as compared to before [*M* = 27.62, *SD* = 8.86, *t*_(25)_ = 9.50, *p* < 0.001]. Mean overall performance rating (i.e., self-perceived overall success in following instructions) was *M* = 79.62% (*SD* = 8.82; Range: 60–90).

### ERP amplitudes

#### N1

**Figure 5:**
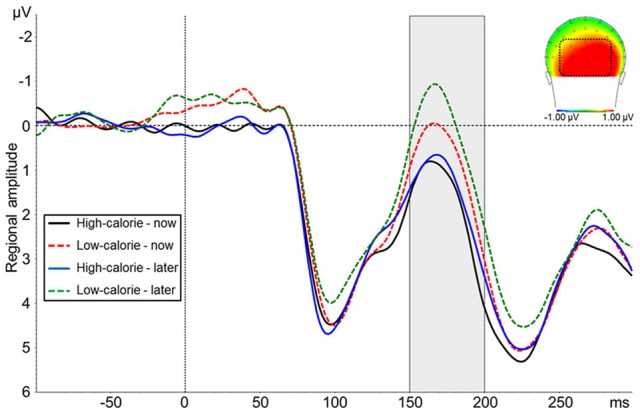
**Mean amplitude of pooled ERPs of a posterior cluster (PO9, PO7, PO3, POz, PO4, PO8, PO10, O1, Oz, O2)**. The headplot shows the difference between trials with high-calorie minus low-calorie food pictures in a time window between 150–200 ms.

The ANOVA on N1 amplitudes revealed a significant main effect for picture type [*F*_(1, 24)_ = 22.88, *p* < 0.001, η^2^_*p*_ = 0.49] indicating a more negative amplitude in response to LC food pictures (*M* = 0.29 μV, *SD* = 4.66) as compared to HC food pictures (*M* = 1.39 μV, *SD* = 4.94; Figure 5). There was no main effect for perspective [*F*_(1, 24)_ = 3.97, *ns*] and no interaction perspective × picture type [*F*_(1, 24)_ = 1.25, *ns*].

#### LPP

**Figure 6:**
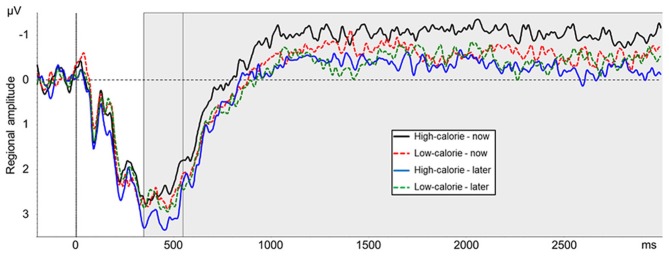
**Mean amplitude of pooled ERPs of a centro-parieto-occipital cluster (CP2, CP4, CP6, P2, P4, P6, PO4)**. Gray shaded boxes mark time windows between 350–550 ms and 550–3000 ms.

The main effect for picture type was not significant [*F*_(1, 24)_ = 0.89, *ns*, η^2^_*p*_ = 0.04]. A main effect for perspective [*F*_(1, 24)_ = 5.78, *p* < 0.05, η^2^_*p*_ = 0.19] was modulated by picture type [interaction perspective × picture type: *F*_(1, 24)_ = 5.17, *p* < 0.05, η^2^_*p*_ = 0.18]. *Post-hoc t*-tests indicated that LPP amplitude was more positive in the HC-LATER condition (*M* = 3.17 μV, *SD* = 2.59) than in all other conditions [HC-NOW: *M* = 2.45 μV, *SD* = 2.17, *t*_(24)_ = 2.99, *p* < 0.01; LC-LATER: *M* = 2.72 μV, *SD* = 2.20, *t*_(24)_ = 2.16, *p* < 0.05; LC-NOW: *M* = 2.64 μV, *SD* = 2.38, *t*_(24)_ = 2.40, *p* < 0.05; Figure 6]. LPP amplitudes in the HC-NOW, LC-NOW, and LC-LATER conditions did not differ from each other [all *t*s_(24)_ < 1.23, *ns*]^1^.

#### Slow wave

The main effect for picture type was not significant [*F*_(1, 24)_ = 1.14, *ns*, η^2^_*p*_ = 0.05]. A main effect for perspective [*F*_(1, 24)_ = 8.18, *p* < 0.01, η^2^_*p*_ = 0.25] was modulated by picture type [interaction perspective × picture type: *F*_(1, 24)_ = 4.26, *p* < 0.05, η^2^_*p*_ = 0.15]. *Post-hoc t*-tests indicated that the slow wave amplitude was less positive in the HC-NOW condition (*M* = −0.82 μV, *SD* = 1.68) than in all other conditions [HC-LATER: *M* = −0.04 μV, *SD* = 1.68, *t*_(24)_ = 3.20, *p* < 0.01; LC-LATER: *M* = −0.22 μV, *SD* = 1.68, *t*_(24)_ = 3.20, *p* < 0.01; LC-NOW: *M* = −0.37 μV, *SD* = 1.78, *t*_(24)_ = 2.83, *p* < 0.01; Figure 6]. Slow wave amplitudes in the HC-LATER, LC-LATER, and LC-NOW conditions did not differ from each other [all *t*s_(24)_ < 1.48, *ns*]^2^.

### Correlations between ERP amplitudes and self-report measures

#### N1

Amplitudes did not correlate with any of the self-report measures.

#### LPP

**Figure 7:**
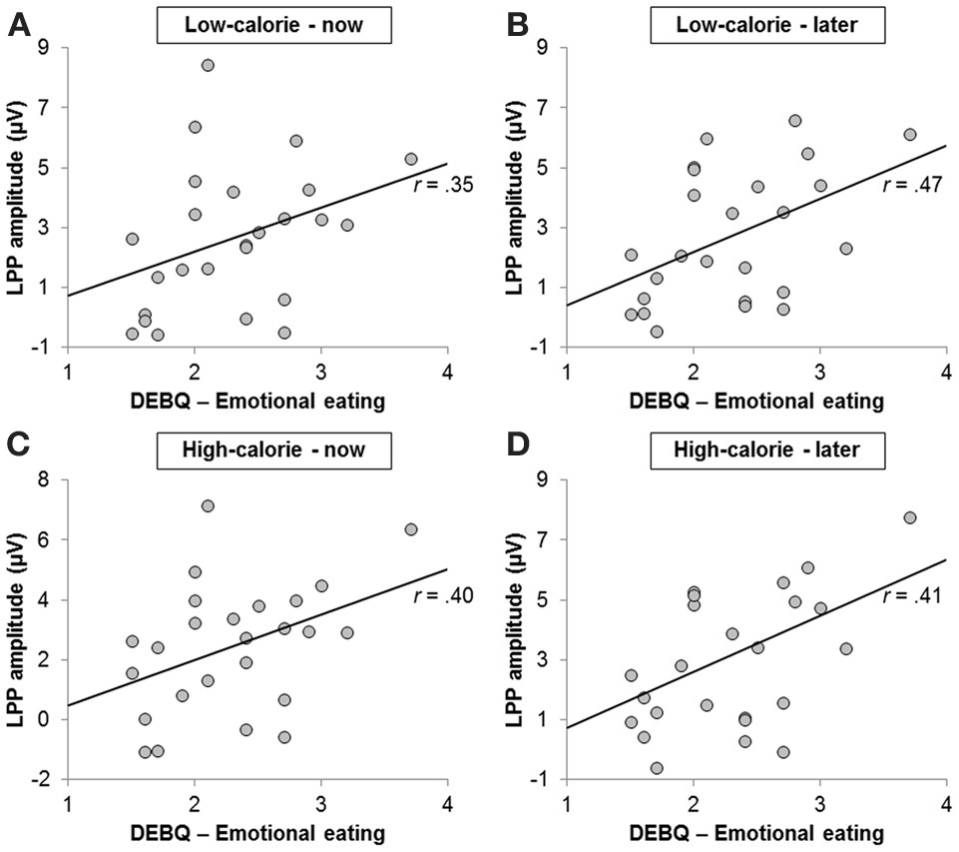
**Scatter plots showing correlations between scores on the emotional eating subscale of the Dutch Eating Behavior Questionnaire and Late Positive Potential amplitude in the (A) low-calorie—now, (B) low-calorie—later, (C) high-calorie—now, and (D) high-calorie—later condition**.

LPP amplitudes pooled across all conditions were positively correlated with the emotional eating subscale of the DEBQ (*r* = 0.42, *p* < 0.05; Figure 7) and with craving ratings in the HC-LATER condition (*r* = 0.57, *p* < 0.01; LPP amplitudes broken down by condition: HC-LATER *r* = 0.50, *p* < 0.05, LC-NOW *r* = 0.54, *p* < 0.01, HC-NOW *r* = 0.54, *p* < 0.01, LC-LATER *r* = 0.63, *p* < 0.01). LPP amplitudes did not correlate with craving ratings in any other condition (all *r*s < 0.27, *ns*).

#### Slow wave

Amplitudes in the HC-NOW condition were negatively correlated with the RS (*r* = −0.40, *p* < 0.05). Craving ratings in the LC-LATER condition were positively correlated with amplitudes in the HC-NOW (*r* = 0.40, *p* < 0.05) and LC-NOW condition (*r* = 0.47, *p* < 0.05).

## Discussion

The current study investigated the electrophysiological correlates of food-cue processing during craving regulation. For this purpose, we used pictures of high- vs. low-calorie foods which did not differ in normative palatability ratings and physical characteristics. A first finding was that early neural processing differed between pictures of high- and low-caloric foods and was not modulated by cognitive regulation. LC food pictures elicited a larger negativity than HC food pictures at occipital sites between 150 and 200 ms after stimulus onset. This finding replicates prior studies with regard to time range, polarity and topography (Toepel et al., 2009; Frank et al., 2010) and suggests that visual areas support an early attentional discrimination of caloric content of food, possibly due to its relevance for survival. In addition, a main effect of calorie content could be observed for LPP amplitude in a left-hemispheric centro-parietal cluster (see Footnote 1), replicating that differences in the processing of HC and LC food-cues can also be found in later stages of food-cue processing and brain areas other than the occipital lobe (Toepel et al., 2009; Frank et al., 2010). It appears that the brain tracks the energetic value of food images and it is unlikely that differences emerged as a result of palatability of those foods or physical characteristics of the pictures. Yet, pictures of HC foods often display processed foods and prepared meals while pictures of LC foods often include whole foods which need to be prepared for eating. Thus, picture categories may not have differed in visual complexity but maybe in complexity with regard to content and food composition. Future studies are needed in which HC and LC food pictures are matched in this regard.

Overall, participants reported higher food craving after the task as compared to before which is a common finding when individuals are engaged in a cognitive task involving appealing food pictures (e.g., Meule et al., 2012c). Evaluations of food craving after each trial, however, revealed that perspective effects depended on caloric content. As expected, for HC foods, thinking about the long-term consequences decreased craving. For LC foods, by contrast, thinking about the long-term consequences *in*creased craving. This finding offers some interesting avenues of intervention toward inducing healthier food choices through cognitive strategies. Furthermore, the current study supports prior findings by showing that HC foods are not necessarily craved and perceived as appealing, but that contextual frames can easily influence if HC foods are associated with palatable or unhealthy (Roefs et al., 2006).

LPP amplitudes reflect the dynamic changes of attentional-motivational processing. When participants were instructed to think about the long-term effects of eating high caloric foods, craving was lowest, and LPP amplitude in a right-hemispheric centro-parietal cluster was enlarged relative to all other conditions. This finding is surprising on first sight because heightened LPP amplitude is considered to reflect motivated attention toward rewarding stimuli like drugs or food, possibly indicating increased craving for those cues (Field et al., 2009; Blechert et al., 2010; Svaldi et al., 2010; Littel et al., 2012; Nijs and Franken, 2012). Indeed, self-rated craving correlated with LPP amplitudes in all conditions. However, affective picture processing research suggests that LPP amplitude is driven by arousal regardless of valence (Olofsson et al., 2008; Hajcak et al., 2010). Thus, in the present data increased LPP amplitude in the HC-LATER condition could also reflect arousal, as it arises from negative thoughts about averse long-term effects of eating HC foods.

In accordance with ERP studies on craving regulation in substance abuse (Littel and Franken, 2011) we followed-up the development of LPP amplitudes during later processing stages. Between 550 and 3000 ms after stimulus onset, the slow wave amplitude was less positive when participants were instructed to think about the immediate, pleasurable effects of eating HC foods (i.e., in the HC-NOW condition) as compared to all other conditions, possibly due to a late motivational engagement with the appetitive value of HC foods. Amplitude in the HC-LATER condition, by contrast, which had been increased in the LPP time range was reduced to the level of the LC conditions during the slow wave time window. Although craving ratings were not systematically correlated with ERP amplitudes in this time range, we would speculate that this pattern of ERP amplitude changes may indicate successful regulation of craving. This interpretation would mirror results of the study by Littel and Franken (2011) in which the application of cognitive craving regulation strategies initially led to increased LPP amplitudes in response to smoking pictures, but to attenuated amplitudes in a late LPP (slow wave) time window.

Finally, scores on the emotional eating subscale of the DEBQ were positively correlated with LPP amplitudes across all conditions. This suggests that individuals who habitually exhibit emotional eating behavior may show a chronic vigilance and enhanced attentional processing of food in general due to its relevance for emotion regulation and behavioral control. While this interpretation is limited by the absence of a neutral control condition in the present study, it is in line with a recent study in which heightened LPP amplitudes in response to food-cues could also be observed in high emotional eaters as compared to low emotional eaters (Blechert et al., in revision).

The current study has several limitations. First, although most participants indicated after the task that they were able to effectively use the regulation strategies, it would have been beneficial to assess regulation success after each trial, as has been done in other studies (e.g., Hollmann et al., 2012). This would allow for condition-wise analyses of ERPs in relation to regulation success. Second, we did not have a neutral condition, i.e., showing pictures of neutral objects or presenting the food-cues without a craving regulation instruction. While some studies found that successful down-regulation of negative emotions or craving may reduce late LPP to the level of neutral images (e.g., Littel and Franken, 2011; Thiruchselvam et al., 2011), this effect cannot be evaluated in the current study. However, the instructional frame would not work sensibly with non-edible images. Third, HC and LC food pictures did not differ in palatability based on normative ratings. While this is an advantage because any ERP differences between categories are probably due to calorie content, future studies may use bland food items as a further control condition. Fourth, all participants were presented with the very same food pictures. Naturally, there are individual differences in food preference and, therefore, specific kinds of food pictures trigger different responses in different individuals. Likewise, a recent study emphasizes the importance of using idiosyncratic food-cues, i.e., determining individually craved foods, in craving regulation studies (Giuliani et al., 2013). Yet, an overall increase in craving across the task indicated that most participants experienced the food stimuli as appealing. Finally, participants were moderately hungry in the current study. As a result, effects of homeostatic hunger might have attenuated differential correlations of eating behavior traits like emotional or restrained eating which are reflective of the more hedonic aspects of hunger (cf. Lowe and Butryn, 2007; Lowe, 2009). Thus, future studies may benefit from including conditions in which participants are either hungry or satiated while regulating their cravings.

Several future directions appear promising. First, future studies could examine conditions that involve either compromised or pathologically enhanced craving regulation (e.g., patients with bulimia nervosa/binge eating disorder and anorexia nervosa, respectively). Second, external validity might be enhanced by incorporating actual eating into the task, for example by manipulating food availability (e.g., Werthmann et al., 2013). Some recent studies found differences in psychophysiological responses to food stimuli when foods that were immediately available to eat were contrasted to foods that were unavailable to eat (Blechert et al., 2010; Rejeski et al., 2010). For instance, available foods elicited an elevated hemodynamic response in reward-related brain areas as compared to unavailable foods (Richter et al., 2013). Thus, effects of craving regulation might interact with actual availability of the displayed foods and regulation effects observed in available conditions would probably be more representative of daily life eating situations. Finally, we investigated only one strategy for the downregulation of craving but future research might pit several strategies against each other (cf. Giuliani et al., 2013; Yokum and Stice, 2013) to inspire the development of specific and evidence based treatments. Distraction, for example, was shown to impact emotional processing earlier than reappraisal (e.g., Thiruchselvam et al., 2011) and might modulate even the early stages of attentional processing that were immune to reappraisal in the present study.

To conclude, subsequent to an early processing stage, reflecting attentional analysis of energy content, both neural and experiential food-cues responses are modulated by cognitive regulation. Mediated by a dynamic neural activation pattern that might have engaged both aversive imagery and appetitive processing, experienced cravings were influenced in an advantageous direction: low calorie foods were craved more and high calorie foods were craved less under a long-term perspective. Thus, habitually using such cognitive strategies might result in healthier food choice and eating behaviors.

## Author contributions

Adrian Meule and Jens Blechert designed this study and performed data analyses. Adrian Meule wrote the first draft of the manuscript. Jens Blechert and Andrea Kübler revised the manuscript for content and approved the final version of the manuscript.

### Conflict of interest statement

The authors declare that the research was conducted in the absence of any commercial or financial relationships that could be construed as a potential conflict of interest.

## Footnotes

^1^We also explored the corresponding cluster on the left hemisphere (CP1, CP3, CP5, P1, P3, P5, PO3) and found a significant main effect for picture type [*F* (1, 24) = 8.69, *p* < 0.01, η^2^_*p*_ = 0.27] indicating higher LPP amplitude in response to HC food pictures (*M* = 4.27, *SD* = 2.26) relative to LC food pictures (*M* = 3.78, *SD* = 2.00). There was neither a main effect for perspective [*F*_(1, 24)_ = 0.39, *ns*, η^2^_*p*_ = 0.03] nor an interaction perspective × picture type [*F*_(1, 24)_ = 0.81, *ns*, η^2^_*p*_ = 0.00].

^2^We also explored the corresponding cluster on the left hemisphere (CP1, CP3, CP5, P1, P3, P5, PO3). Both main effects as well as the interaction were not significant [all *F* s_(1, 24)_ < 1.81, *p* > 0.19, η^2^_*p*_ < 0.08].
